# Virulence of *Klebsiella pneumoniae* Isolates Harboring *bla*_KPC-2_ Carbapenemase Gene in a *Caenorhabditis elegans* Model

**DOI:** 10.1371/journal.pone.0067847

**Published:** 2013-07-02

**Authors:** Jean-Philippe Lavigne, Gaelle Cuzon, Christophe Combescure, Gisèle Bourg, Albert Sotto, Patrice Nordmann

**Affiliations:** 1 Institut National de la Santé et de la Recherche Médicale, U1047, Université Montpellier 1, UFR de Médecine, Nîmes, France; 2 Laboratoire de Bactériologie, CHU Carémeau, Nîmes, France; 3 Service de Bactériologie-Virologie-Hygiène, Institut National de la Santé et de la Recherche Médicale U914, AP-HP Bicêtre, Université Paris Sud, Kremlin-Bicêtre, France; 4 Division of Clinical Epidemiology, University of Geneva, Geneva, Switzerland; Charité-University Medicine Berlin, Germany

## Abstract

*Klebsiella pneumoniae* carbapenemase (KPC) is a carbapenemase increasingly reported worldwide in *Enterobacteriaceae*. The aim of this study was to analyze the virulence of several KPC-2-producing *K. pneumoniae* isolates. The studied strains were (i) five KPC-2 clinical strains from different geographical origins, belonging to different ST-types and possessing plasmids of different incompatibility groups; (ii) seven transformants obtained after electroporation of either these natural KPC plasmids or a recombinant plasmid harboring only the *bla*_KPC-2_ gene into reference strains *K. pneumoniae* ATCC10031/CIP53153; and (iii) five clinical strains cured of plasmids. The virulence of *K. pneumoniae* isolates was evaluated in the *Caenorhabditis elegans* model. The clinical KPC producers and transformants were significantly less virulent (LT50: 5.5 days) than *K. pneumoniae* reference strain (LT50: 4.3 days) (p<0.01). However, the worldwide spread KPC-2 positive *K. pneumoniae* ST258 strains and reference strains containing plasmids extracted from *K. pneumoniae* ST258 strains had a higher virulence than KPC-2 strains belonging to other ST types (LT50: 5 days vs. 6 days, p<0.01). The increased virulence observed in cured strains confirmed this trend. The *bla*_KPC-2_ gene itself was not associated to increased virulence.

## Introduction

*Klebsiella pneumoniae* carbapenemases (KPC) are broad-spectrum β-lactamases, which confer resistance to all β-lactam molecules, including carbapenems. Those plasmid-encoded enzymes are identified mostly in *K. pneumoniae* [1]–[3]. KPC producers have been increasingly reported worldwide [1]. Eleven KPC variants (KPC-2 to KPC-12) are known differing by a few amino acids changes, KPC-2 being the most frequent KPC-type enzyme. KPC-associated enterobacterial infections do not seem to be specific to sites, organs, or tissues: most are either systemic infections, occurring in patients with multiple invasive devices or urinary tract infections without an indwelling catheter, particularly in immunocompromised patients [4], [5]. No specific virulence factors have been identified as being associated to KPC-producing strains [1]. Risk factors associated with the acquisition of KPC-producing bacteria included prolonged hospitalisation, intensive care unit hospitalisation, invasive devices, immunosuppression, and multiple antibiotic agents before initial culture as known for many other multidrug resistance Gram-negative bacilli [4], [6].

The present study was aimed to evaluate the virulence of several *bla*_KPC-2_ producing *K. pneumoniae* strains using *Caenorhabditis elegans* model of infection.

## Materials and Methods

### Bacterial Strains

The studied *K. pneumoniae* strains are listed in Table 1. Five clinical KPC-2-producing *K. pneumoniae* strains were studied (origin of isolates and MLST type are indicated in brackets) (Table 1): YC (USA, ST-258), KN2303 (Colombia, ST-337), KN633 (Colombia, ST-338), A28006 (Brazil, ST-11) and 475 (Israel, ST-277) [7]–[11]. Virulence of these strains for *C. elegans* was compared with *K. pneumoniae* strain ATCC10031/CIP 53153. *E. coli* strain OP50, an avirulent strain corresponding to food for lab worms, was used as the negative control in the *C. elegans* model.

**Table 1:** Main characteristics of the strains and plasmids used in this study.

Strain or plasmid	Source	Plasmid gene contents	ST	Virulence traits	Reference
*K. pneumoniae* ATCC10031/CIP53153	FDA	–	–	*fimH-1, mrkD, kpn, ycfM, entB, irp1, irp2, ybtS, fyuA, iutA, CPS*	ATCC collection
*K. pneumoniae* KN2303	Clinical (Colombia)	*bla*_KPC-2_	337	*fimH-1, mrkD, kpn, ycfM, entB, CPS*	7
*K. pneumoniae* KN633	Clinical (Colombia)	*bla*_KPC-2_ *bla*_TEM-1_ *bla*_CTX-M-12_	338	*fimH-1, mrkD, kpn, ycfM,* *entB, CPS*	7
*K. pneumoniae* YC	Clinical (USA)	*bla*_KPC-2_ *bla*_TEM-1_ *bla*_OXA-9_	258	*fimH-1, mrkD, kpn, ycfM, entB, CPS*	8
*K. pneumoniae* A28006	Clinical (Brazil)	*bla*_KPC-2_ *bla*_TEM-1_ *bla*_CTX-M-2_	11	*fimH-1, mrkD, kpn, ycfM, entB, CPS*	9
*K. pneumoniae* 475	Clinical (Israel)	*bla*_KPC-2_ *bla*_CTX-M-15_	277	*fimH-1, mrkD, kpn, ycfM, entB, CPS*	10
CIP53153+ pBC2303	Transformant with plasmid extracted from KN2303	*bla*_KPC-2_	–	–	This study
CIP53153+ pBC633	Transformant with plasmid extracted from KN633	*bla*_KPC-2_	–	–	This study
CIP53153+ pNYC	Transformant with plasmid extracted from YC	*bla*_KPC-2_	–	–	This study
CIP53153+ pA28006	Transformant with plasmid extracted from A28006	*bla*_KPC-2_	–	–	This study
CIP53153+ p475	Transformant with plasmid extracted from 475	*bla*_KPC-2_	–	–	This study
CIP53153+ pBKCMV	Transformant with a plasmid wich was used for the cloning experiment	–	–	–	This study
CIP53153+ pRYC-1	Transformant with a plasmid containing the *bla*_KPC-2_ gene	*bla*_KPC-2_	–	–	This study
KN2303– pBC2303	Plasmid cure derivative strain	–	337	–	This study
KN633– pBC633	Plasmid cure derivative strain	–	338	–	This study
YC – pNYC	Plasmid cure derivative strain	–	258	–	This study
A28006– pA28006	Plasmid cure derivative strain	–	11	–	This study
475– p475	Plasmid cure derivative strain	–	277	–	This study
pBKCMV	Vector		–	–	11
pRYC-1	pBKCMV-derivated recombinant plasmid carrying *bla*_KPC-2_	*bla*_KPC-2_	–	–	11

Bacterial strains were cultured onto Trypticase soya (TS) agar plates at 37°C under a 5% CO_2_ atmosphere. Bacterial isolates were stored at –80°C.

### Transformation

Plasmids pBC633 (12 kb), pBC2303 (75 kb, IncN), pNYC (80 kb, IncFII), pA28006 (12 kb, IncL/M) and p475 (80 kb, IncN) encoding *bla*_KPC-2_ gene were extracted from the clinical *K. pneumoniae* KN633, KN2303, YC, A28006 and 475 isolates respectively, and purified using the Qiafilter Plasmid Midi kit (Qiagen, Courtaboeuf, France) following the manufacturer recommendations. These plasmids were then electroporated into competent *K. pneumoniae* strain ATCC10031/CIP 53153. Transformants were selected on Luria Broth agar plates containing 0.5 mg/L of imipenem. Plasmid profiles of transformants were checked after their extraction as previously described [11].

The construction of the recombinant plasmid pRYC-1 that contained a *bla*_KPC-2_ gene into cloning vector pBKCMV has been previously described [11]. This recombinant plasmid was extracted from the recipient strain, purified and electroporated into competent *K. pneumoniae* ATCC10031/CIP 53153. Transformants were selected on Luria Broth agar plates containing 0.5 mg/L of imipenem. The cloning vector pBKCMV which did not contain a *bla*_KPC-2_ gene was also electroporated into competent *K. pneumoniae* ATCC10031/CIP 53153 using kanamycin as selection agent.

### Plasmid Curing

The curing of plasmids of each clinical *K. pneumoniae* strain was performed by successive bacterial subcultures at 44°C as described previously [12]. The loss of the plasmids was visualized by agarose gel electrophoresis and confirmed by negative PCR results for the selected plasmid genes including *bla*_KPC-2_ gene (Table 1).

### Resistance Traits

Susceptibility to β-lactams in particularly to carbapenems, fluoroquinolones, tigecycline, cotrimoxazole, aminoglycosides, and colistin was determined by either the E-test (bioMérieux, Marcy l’Etoile, France) or by the agar disk diffusion method. MIC determination was performed and interpreted as recommended by the Clinical and Laboratory Standards Institute (CLSI), as updated in 2012. The *bla*_KPC_, *bla*_CTX-M_, *bla*_SHV_, *bla*_TEM_, *bla*_OXA-1/9_-like genes were searched by PCR and characterized as described previously [1], [11].

### Genotypic and Phenotypic Determination of Virulence Factors

The *K. pneumoniae* isolates were tested by PCR for the presence of a panel of genes encoding known virulence factors. Methods used to amplify *fimH-1* (mannose-specific adhesin subunit of type 1 fimbriae), *mrkD* (mannose-specific adhesin subunit of type 3 fimbriae), *kpn* (FimH-like adhesin), *ycfM* (outer membrane lipoprotein), *iroN* (catecholate siderophores receptor), *entB* (enterobactin biosynthesis), *iutA* (aerobactin), *fyuA* (yersiniabactin receptor), *traT* (serum resistance), *irp1, irp2, ybtS* (yersiniabactin biosynthesis), *rmpA* (regulator of mucoid phenotype A), *magA* (mucoviscosity-associated gene A), *hlyA* (hemolysin), and *cnf1* (cytotoxic necrotizing factor-1) have been described elsewhere [13].

The presence of a capsule was determined by staining with India ink. On a slide, a drop of bacterial suspension was mixed with the stain. By light microscopy, the capsule appears as a clear area between the colored background and the stained bacterial body.

### Nematode Killing Assay

The *C. elegans* model has been developed to study host-pathogen interactions. In this test, the studied bacteria is presented as food to the nematodes instead of *E. coli* strain OP50, an avirulent strain, that is their usual food in the lab. Ingestion of the bacteria studied by the worms results in an infection and ultimately the killing death of the worms [14]. The time required for bacteria to kill the worms compared with life duration observed when the worms are fed with *E. coli* strain OP50, is an indirect marker of virulence potential of the studied bacteria. The *C. elegans* infection assay was carried out as described by Lavigne *et al*. [14] using the Fer-15 mutant line, which has a temperature sensitive fertility defect. To synchronize the growth of worms, eggs were collected using the hypochlorite method. Overnight cultures in nematode growth medium (NGM) of *K. pneumoniae* strains were harvested by centrifugation, washed once and suspended in phosphate buffered saline solution (PBS) at pH 7.0 at a concentration of 10^5^ CFU/ml. NGM agar plates were inoculated with 10 µl of each strain and incubated at 37°C for 8 to 10 h. Plates were allowed to cool to room temperature and seeded with L4 stage worms (20–30 per plate). Plates were then incubated at 25°C and scored each day for live worms under a stereomicroscope (Leica MS5) [14]. At least three replicates repeated five times were performed for each selected clone. Lethal time 50% (LT50) corresponded to time (hours) required to kill 50% of the nematode population. The results are representative of five independent assays for each group of strains.

### *In vitro* Strain Growth

Bacteria were grown in LB broth and NGM broth. An aliquot (1 mL) of an overnight culture of each strain was transferred in 99 mL and cultured at 37°C under shaking conditions. Bacterial growth was monitored by change of the optical density at 620 nm (OD_620_) and determined over a period of 24 h. Growth curves were performed in triplicate for each strain and the OD_620_ measurements were averaged for each time point in order to plot growth curves.

### Bacterial Ingestion in Worms

*In vivo* experiments consisted of measuring the number of bacteria within the *C. elegans* digestive tract 72 h after ingestion as described by Garsin *et al.* [15]. Five *C. elegans* were picked at 72 h, and washed twice in 4 µl drops of M9 medium. The nematodes were placed in a 1.5 ml Eppendorf tube containing 20 µl of M9 medium with 1% Triton X-100 and were mechanically disrupted by using a pestle. The volume was adjusted to 50 µl with M9 medium containing 1% Triton X-100 which was diluted and plated on TS agar containing 50 mg/L of ampicillin. At least three replicates were performed for each bacterial challenge. Antibiotic susceptibility of each strain recovered from the digestive tract of the nematodes was determined by the disk agar diffusion method and compared to that of the corresponding strain given as food to the nematodes.

### Bacterial Lawn Avoidance Assay

Small lawns of the different studied strains were cultured on NGM plates overnight at 25°C. Approximatively 30 young adult worms grown on OP50 were put in the centre of each bacteria lawn. The number of animals on each lawn was counted after 4, 8 and 16 h as previously described [16]. The results were explained in percent occupancy corresponding to the number of worms in the bacterial lawn/the total number of worms. The experiments were performed in triplicate.

### Statistical Analysis

In order to perform pairwise comparison between two different strains, we used a log rank test. The Kruskall–Wallis test was used to compare the *in vitro* and *in vivo* bacterial growth of the different strains. A *p* value ≤0.05 given by SPSS 6.1.1 (SAS Institute Inc, Cary, NC, USA) was considered as reflecting statistical significance.

## Results

### Antibiotic Susceptibility

As with most clinical KPC producers [1], the strains were susceptible to colistin and tigecycline. *K. pneumoniae* KN2303 and *K. pneumoniae* KN633 were susceptible to all tested aminoglycosides and fluoroquinolones, *K. pneumoniae* YC was susceptible only to gentamicin, *K. pneumoniae* A28006 was susceptible to aminoglycosides, and *K. pneumoniae* 475 was only susceptible to amikacin, levofloxacin and ciprofloxacin.

The isolates contained several plasmids differing in structure and size. In each case, a single plasmid harboring the *bla*_KPC-2_ gene was electroporated into the same *K. pneumoniae* reference strain ATCC10031/CIP 53153. The antibiotic susceptibilities of the transformants are shown in Table 2. PCR and plasmid extraction analysis confirmed the presence of a single plasmid harboring a *bla*_KPC-2_ gene in those transformants. Transformants were resistant to at least ceftazidime and carbapenems, which was related to the expression of the carbapenemase KPC-2 (Table 2). In most of the cases, co-resistance to non-ß-lactam antibiotics was not observed for those transformants. The only exception was for transformant p475, which showed resistance to aminoglycosides (gentamicin and tobramycin) and cotrimoxazole. Conversely, strains cured of plasmids had lost resistance to extended-spectrum cephalosporins and carbapenems (data not shown).

**Table 2:** Antibiotic susceptibility of the *K. pneumoniae* transformant strains.

	MIC (µg/ml)					
Antibiotics	*K. pneumoniae* CIP53153	*K. pneumoniae* CIP53153+ pNYC	*K. pneumoniae* CIP53153+ pA28006	*K. pneumoniae* CIP53153+ p475	*K. pneumoniae* CIP53153+pBC633	*K. pneumoniae* CIP53153+pBC2303
Amoxicillin	96	≥256	≥256	≥256	≥256	≥256
Amoxicillin+CLA	2	≥256	≥256	≥256	≥256	≥256
Ticarcillin	96	≥256	≥256	≥256	≥256	≥256
Ticarcillin+CLA	1.5	≥256	≥256	≥256	≥256	≥256
Piperacillin	2	≥256	≥256	≥256	≥256	≥256
Piperacillin+TZB	0.094	≥256	≥256	≥256	≥256	≥256
Ceftazidime	0.094	12	16	24	32	16
Imipenem	0.19	8	8	12	8	8
Ertapenem	0.016	4	3	4	4	4
Tigecycline	0.125	0.125	0.125	0.125	0.125	0.125
Gentamicine	<2	<2	<2	>4	<2	<2
Tobramycin	<2	<2	<2	>4	<2	<2
Amikacin	<2	<2	<2	<2	<2	<2
Ciprofloxacin	<0.5	<0.5	<0.5	<0.5	<0.5	<0.5
Cotrimoxazole	<1	<1	<1	>4	<1	<1
Colistin	<2	<2	<2	<2	<2	<2

### Virulence

The distribution of virulence factors is shown in Table 1. The different adhesins-encoding genes were present in all the isolates. The siderophore gene *entB* was also present in 100% of the strains. However, only the reference strain ATCC10031/CIP53153 harbored aerobactin genes (*irp1, irp2, ybtS*). None of our isolates carried *rmpA*, *magA, cnf1*, and *hlyA* genes.

The results of nematode killing assays are presented in Table 3.

**Table 3:** Letal Time 50% (LT50) of *Caenorhabditis elegans* infected by clinical and transformant *K. pneumoniae* strains.

StrainCharacteristics of strain	LT50 in days		(Mean ±se)	*p*			
				ATCCvs other strains	clinical vs transformants/derivatives	475,YC,A28006 (clinical/mutants)vs 2303,633 (clinical/mutants)	pRYC-1 vs other strain
ATCC10031/CIP53153		Reference	4.3±0.2	–			NS
KN2303		Clinical isolate	5.7±0.3	<0.001	NS	<0.01	<0.001
KN633		Clinical isolate	6.0±0.5	<0.001	NS	<0.01	<0.001
YC		Clinical isolate	5.3±0.2	<0.001	NS	<0.01	<0.001
A28006		Clinical isolate	5.0±0.5	<0.001	NS	<0.01	<0.001
475		Clinical isolate	5.3±0.2	<0.001	NS	<0.01	<0.001
CIP53153+ pBC2303		Transformant	5.9±0.1	<0.001	NS	<0.01	<0.001
CIP53153+ pBC633		Transformant	6.3±0.2	<0.001	NS	<0.01	NS
CIP53153+ pNYC		Transformant	5.6±0.4	<0.001	NS	<0.01	<0.001
CIP53153+ pA28006		Transformant	5.5±0.5	<0.001	NS	<0.01	<0.001
CIP53153+ p475		Transformant	5.5±0.5	<0.001	NS	<0.01	<0.001
pBk-CMV		Transformant	4.6±0.3	NS	–	–	–
pRYC-1		Transformant	6.6±0.4	<0.001	–	–	<0.001
KN2303– pBC2303		Plasmid cured derivative	5.0±0.5	<0.001	<0.001	<0.01	<0.01
KN633– pBC633		Plasmid cured derivative	5.0±0.5	<0.001	<0.001	<0.01	<0.01
YC – pNYC		Plasmid cured derivative	4.7±0.3	NS	<0.001	<0.01	<0.01
A28006– pA28006		Plasmid cured derivative	4.3±0.3	NS	<0.001	<0.01	<0.01
475– p475		Plasmid cured derivative	4.5±0.5	NS	<0.001	<0.01	<0.01
OP50		Avirulent, nematode food	8.2±0.3	<0.001	–	–	–

LT50 corresponds to the time for half of the worms to die. The results are representative of at least five independent trials for each group of strains.

The clinical KPC-producing strains were significantly less virulent (LT50 varied between 5.0 to 6.0 days ±0.5) than reference strain ATCC10031/CIP 53153 (LT50: 4.3 days ±0.2) (p<0.001), but significantly more virulent than the avirulent OP50 (LT50: 8.2 days ±0.3) (p<0.001). Nematode killing results allow us to constitute two groups of clinical KPC strains. A first group included *K. pneumoniae* YC, 475 and A28006 (ST-258 type and related ST type) with a mean survival time of 5 days (±0.5) and a second group of *K. pneumoniae* KN2303 and KN633 (non-ST-258 type), with a mean survival time of 6 days (±0.5). The difference in virulence between both groups was significant (p<0.01). Then, the five plasmids (pBC633, pBC2303, pNYC, pA28006 and p475) were electroporated into competent *K. pneumoniae* ATCC10031/CIP 53153. Comparison between the virulence of clinical strains and the corresponding transformants showed no significant difference (p = 0.4, 0.10, 0.96, 0.81 and 0.13 for YC, A28006, 2303, 633 and 475, respectively). The three transformants (pNYC, p475 and pA28006) strains were significantly more virulent than that of the two transformants (pBC633 and pBC2303) strains (LT50 varied between 5.5 to 5.6 vs 5.9 to 6.3 days ±0.5, respectively) (p<0.001) showing an increased virulence strains harboring KPC plasmids from strains belonging to ST-type 258.

The five transformant strains remained significantly less virulent than the recipient ATCC10031/CIP 53153 strain (LT50 varied between 5.5 and 6.3 vs 4.3 days ±0.5, respectively) (p<0.001) suggesting that the introduction of the plasmid-mediated *bla*_KPC-2_ gene decreased the virulence of the reference strain (Table 3). To support the direct effect of the plasmid in the bacterial virulence, the virulence of plasmid cured strains was determined. The cured strains were all significantly more virulent than the corresponding parental strains (LT50 varied between 4.3 to 5.0 vs 5.0 to 6.0 days ±0.5, respectively) (p<0.001).

To analyze the role of the *bla*_KPC-2_ gene itself, virulence of a same strain (ATCC10031/CIP 53153) harboring a recombinant plasmid containing the *bla*_KPC-2_ gene (pRYC-1) was analysed and compared to that of the same plasmid without the *bla*_KPC-2_ gene. Strain harboring pRYC-1 was significantly less virulent than the reference strain and the three most virulent clinical strains (YC, 475 and A28006) (LT50 varied between 6.6 vs 5.5 and 5.6 days ±0.5, respectively) (p<0.001). In addition, introduction of the plasmid pBK-CMV (cloning plasmid) without the *bla*_KPC-2_ gene did not modify the virulence of the strain (LT50 varied between 4.6 vs 4.3 days ±0.3, respectively) (p = 0.19) (Table 3).

### *In vitro* Bacterial Growth

The growth curves determined in LB medium did not show significant difference between the studied strains and the corresponding transformants. The same results were obtained by culturing the strains in the NGM medium which is used for the nematode assays (data not shown). Thus, the presence or introduction of a KPC-encoding plasmid into reference strain had no impact on the *in vitro* bacterial growth. This also confirms that the *in vivo* results were not biased by growth in NGM medium.

### Bacterial Ingestion

The differences in killing were not due to differences in the survival and proliferation of the strains in the nematode intestine since the bacterial colonization with the different strains in the digestive tract of nematodes measured 72 h after ingestion was 10^6 ^CFU without significant difference (data not shown). The antibiotic susceptibility of the strains obtained after passages in the digestive tract was strictly identical to that of the strains given as food to worms (data not shown).

Moreover, the occupancy of the worms on bacterial lawns was similar for all the strains around 100% without significant difference (Table 4).

**Table 4:** Lawn-leaving behavior of the different strains.

Strains		% occupancy after 16 h
*Control strain*	*K. pneumoniae* ATCC10031/CIP 53153	100 (±1)
*Clinical strains*	*K. pneumoniae* KN2303	98 (±2)
	*K. pneumoniae* KN633	97 (±3)
	*K. pneumoniae* YC	100 (±1)
	*K. pneumoniae* 475	99 (±1)
	*K. pneumoniae* A28006	100 (±1)
*Transformants*	pBC2303	99 (±2)
	pBC633	97 (±4)
	pNYC	99 (±2)
	pA28006	100 (±1)
	p475	100 (±1)
	pBk-CMV	97 (±2)
	pRYC-1	100 (±1)
*Plasmid cure derivatives*	KN2303– pBC2303	100 (±1)
	KN633– pBC633	98 (±2)
	YC – pNYC	98 (±2)
	A28006– pA28006	100 (±1)
	475– p475	100 (±2)

## Discussion

Recent data suggest a relationship between *K. pneumoniae* virulence and drug resistance [17]–[20]. Expression of efflux pumps in *K. pneumoniae* as a mechanism of antibiotic resistance may be associated with expression of novel virulence factors required to resist to innate immune defense mechanisms in nematode and murine models and may be also associated to resistance to the host antimicrobial peptides [17], [18]. The impact of carbapenemase expression on virulence is weak [21]. A recent study suggests that known virulence factors such as K1, K2, and K5 capsular polysaccharides, *rmpA* and the aerobactin gene were absent in KPC-producing isolates and these strains present low virulence in a murine lethality model [21]. In this present study, we have investigated the influence played by different plasmids harboring a single carbapenemase gene, the *bla*_KPC-2_ gene on the bacterial ability to colonize and infect the *C. elegans* nematode. Five clinical isolates from US, Colombia, Israel and Brazil which belonged to different ST clones were studied. In nematode killing assay, the KPC-2 clinical isolates were significantly less virulent (LT50: 5 to 6 days ±0.5) than reference strain ATCC10031/CIP 53153 (LT50: 4.3 days ±0.2) (p<0.001). The analysis of the distribution of virulence factors in each group of strains could explain this observation with the absence of some siderophores genes in KPC isolates. However, we found the presence of the genes encoding adhesins in all the strains that demonstrates a virulence potential of the resistant strains (as also suggested by the results of the strains with cured plasmids). Some other virulence factors are now well established in *K. pneumoniae* strains. K antigen, a capsular polysaccharide, is considered to be an important virulence factor of *K. pneumoniae*. According to these capsular polysaccharides, *K. pneumoniae* can be classified into 77 serological K antigen types. K1 isolated in South East Asia was the predominant serotype causing pyogenic liver abscess. K2 serotype is among the most common capsule types isolated from patients with UTI, pneumonia, or bacteremia. It can be assumed that K2 is the predominant serotype of human clinical isolates worldwide [22], [23]. In KPC clinical strains, these serotypes are absent limiting the effect of this important factor of virulence [21].

Among the KPC-2 clinical strains, two groups of bacterial virulence can be constituted: the first group including clone ST-258 and its single- or double-locus variants, ST-11 and ST-277 with an higher virulence trait than the second group including clones ST-377 and ST-378 (p<0.01). Recent findings indicate the international dissemination of KPC-producing strains of ST-258 type [24]. We showed here that an increase of virulence of KPC-2 producing strains belonging to ST-258 (compared to non-ST-258 strains) may be due to the plasmid type from those KPC-2 producers of the ST-258 type. However if we compared the strains 475 and KN2303 each belonging to one of the different ST groups, they display LT50 pattern while they share the same plasmid type (IncN). This implies that other factors than just plasmids are probably also involved in virulence of KPC-2 producers in this model.

In the aim to complete our investigation, we studied the role of the *bla*_KPC-2_ gene in KPC-2 positive plasmids contained in ST-258 *K. pneumoniae.* Actually its presence decreased virulence of the recombinant strains. These results may be explained either by the natural role of the *bla*_KPC-2_ gene itself or a modulation of expression of chromosome-encoded genes. Some bias due to the fact that the *bla*_KPC-2_ gene was carried on a high-copy vector could not be excluded. Therefore, we believe that other factors expressed by plasmids of the KPC-2 plasmids of ST-258 type strain, such as colonization fitness, survival capability in the nosocomial environment, adherence, biofilm production may explain their increased virulence or/and dissemination. Indeed the nucleotide sequence of plasmids carrying the *bla*_KPC-2_ gene is known in several cases [25], [26]. Different virulence factors have been detected notably in strains of ST-258 type, which harboured a type 4-secretion system gene cluster [25]. The impact of this secretion system inducing a short, rigid pilus releasing virulence factors must be studied to evaluate its real role in *K. pneumoniae* virulence.

In conclusion, this study emphasizes that the key point with regard to the balance between virulence and resistance in the multidrug resistant bacteria studied in this work is at least the type of KPC-2 plasmids.
